# The validity of self-report of eye diseases in participants with vision loss in the National Eye Health Survey

**DOI:** 10.1038/s41598-017-09421-9

**Published:** 2017-08-18

**Authors:** Joshua Foreman, Jing Xie, Stuart Keel, Peter van Wijngaarden, Hugh R. Taylor, Mohamed Dirani

**Affiliations:** 1 Centre for Eye Research Australia, Royal Victorian Eye & Ear Hospital, Melbourne, Australia; 2 Ophthalmology, University of Melbourne, Department of Surgery, Melbourne, Australia; 3 Indigenous Eye Health Unit, Melbourne School of Population and Global Health, The University of Melbourne, Melbourne, Australia

## Abstract

We assessed the validity and reliability of self-report of eye disease in participants with unilateral vision loss (presenting visual acuity worse than 6/12 in the worse eye and equal to or better than 6/12 in the better eye) or bilateral vision loss (presenting visual acuity worse than 6/12 in the better eye) in Australia’s National Eye Health Survey. In total, 1738 Indigenous Australians and 3098 non-Indigenous Australians were sampled from 30 sites. Participants underwent a questionnaire and self-reported their eye disease histories. A clinical examination identified whether participants had cataract, age-related macular degeneration, diabetic retinopathy and glaucoma. For those identified as having unilateral or bilateral vision loss (438 Indigenous Australians and 709 non-Indigenous Australians), self-reports were compared with examination results using validity and reliability measures. Reliability was poor for all four diseases (Kappa 0.06 to 0.37). Measures of validity of self-report were variable, with generally high specificities (93.7% to 99.2%) in all diseases except for cataract (63.9 to 73.1%) and low sensitivities for all diseases (7.6% in Indigenous Australians with diabetic retinopathy to 44.1% of non-Indigenous Australians with cataract). This study suggests that self-report is an unreliable population-based research tool for identifying eye disease in those with vision loss.

## Introduction

Population-based health studies are indispensable to our understanding of the prevalence and causes of disease, and their findings often direct medical research priorities and inform evidenced-based policy on resource allocation^1^. Generally, standardised clinical examinations in which all participants are subjected to the same testing protocol generate the most reliable data^2, 3^. However, logistical constraints and financial costs may render clinical examinations unfeasible, and a large proportion of surveys rely exclusively on participants’ self-report to collect data on medical history, disease diagnosis, and other important health-related information^4–6^.

Research has repeatedly revealed disagreement between self-report of eye disease and the results of clinical examinations in surveys^7, 8^. Studies have attributed this poor awareness of personal eye disease to either a lack of previous diagnoses^9^, or inaccurate recall in those who have been diagnosed resulting from memory failure or poor eye health literacy^8, 10^. A number of surveys including the Beaver Dam Eye Study (BDES)^8^, the Wisconsin Epidemiologic Study of Diabetic Retinopathy (WESDR)^11^, and the Los Angeles Latino Eye Study [LALES]^7, 9^ have demonstrated poor reliability and validity of self-report for age-related macular degeneration (AMD), cataract, glaucoma and diabetic retinopathy (DR), with only 5% to 46% of those identified as having an eye disease, accurately self-reporting their disease. These conditions often require early detection, treatment and management to reduce the risk of vision loss^12^. The notably low rates of disease awareness in these studies illustrates that the use of self-report under-estimates both the prevalence and disability weighting of eye diseases^13^, thereby reducing the effectiveness of such measures for resource allocation to reduce the burden of vision loss.

In Australia, self-report data collected by the Australian Bureau of Statistics (ABS) National Health Surveys (NHS) and the National Survey of Disability, Ageing and Carers (NSDAC) are major sources of population data on eye disease, and are used by the Australian Government to formulate eye healthcare policy^14, 15^. There is some evidence to suggest that self-report of eye diseases by both Indigenous Australians^16^ and non-Indigenous Australians^17, 18^ is unreliable. However, these studies did not conduct thorough agreement analyses and did not report sensitivity, specificity, kappa coefficients and predictive values, suggesting that a more comprehensive analysis is required. Furthermore, previous analyses often were limited to a single disease, had limited geographic or ethnic coverage, or did not investigate risk factors for poor reliability of self-report. Considering that self-report surveys continue to be cited as valuable sources of data in eye disease surveillance in Australia and elsewhere, further investigation of the reliability of this method is warranted.

The National Eye Health Survey (NEHS) collected the first nationally-representative data on the prevalence and causes of both unilateral and bilateral vision loss in Indigenous and non-Indigenous Australians, using both self-report and thorough clinical examinations. This paper assesses the reliability of self-report of major eye diseases in NEHS participants with unilateral or bilateral vision loss by comparing self-report with clinical diagnoses, and presents risk factors for inaccurate self-report. The findings of this study may provide insight into the validity of self-report surveys in Australia as well as the accuracy of current surveillance data on the burden of vision-threatening eye disease.

## Materials and Methods

### Study design and participant sampling

This study received ethical approval from the Royal Victorian Eye and Ear Hospital Human Research Ethics Committee (HREC-14/1199H) and state-based ethics bodies that govern research on Indigenous Australians. All procedures adhered to the tenets of the Declaration of Helsinki. The study design and sampling methodology of the NEHS have been described in detail and published elsewhere^19^. In brief, this study was a nationwide population-based survey that used multistage random-cluster sampling to select population clusters from all remoteness strata in Australia using 2011 Census data^20^. Thirty clusters were selected in total, each containing approximately 50 Indigenous Australians aged 40 years and older and 100 non-Indigenous Australians aged 50 years and older. A younger age criterion was selected for Indigenous participants to reflect the earlier onset and more rapid progression of disease in this population^14^. Trained recruiters went door-to-door in each survey site to recruit participants to the study. Employed personnel and local staff from Aboriginal Medical Services (AMS) assisted in the recruitment of Indigenous Australians in each site.

### Survey questionnaire

All study participants provided written informed consent. A standardised questionnaire was administered by trained interviewers. Interviewers slowly read out questions from a standardised questionnaire stored on mobile tablet computers, and input participants’ verbal answers directly into the questionnaire to be uploaded to a cloud-based online database. The relevant questions were;

1. Personal particulars: name, gender (binary male or female), age and date of birth.
2. Ethnicity; country of birth, whether participants were of Aboriginal or Torres Strait Islander origin, and language spoken at home.
3. Educational attainment: highest level of education (9 item scale from Grade 0 = No education to Grade 8 = Undertaking/completed post graduate study), as well as number of years of education.
4. History of stroke (binary Yes/No)
5. Past ocular history: History of having eyes examined (binary Yes/No), time since last examination (months and years) and type of service provider visited (list of options included optometrist, ophthalmologist, local doctor, nurse, technician, other), history of being diagnosed with any of the following diseases; glaucoma, cataract, diabetic retinopathy, age-related macular degeneration (Yes/No/Unsure). Layman terminology was used to describe each disease if participants were unsure of their meaning. All points of ambiguity were clarified and participants were provided with an opportunity to ask any questions.

### Clinical examination

The clinical examination protocol of the NEHS has been described in detail elsewhere^21^. In summary, a 30-minute examination that involved a series of standardised eye tests was conducted by trained examiners. Presenting distance visual acuity was assessed using a logMAR chart (Brien Holden Vision Institute, Australia) with each eye tested separately. Participants with either bilateral vision loss (presenting visual acuity worse than 6/12 in both eyes) or unilateral vision loss (presenting visual acuity worse than 6/12 in one eye) underwent handheld autorefraction (Nidek Co., LTD, Japan) in the affected eye(s) and best-corrected visual acuity was measured. Binocular near vision was assessed used a CERA Vision Test E Chart (Centre for Eye Research Australia, Australia). Slit-lamp examination was performed using a Keeler PSL One hand-held slit-lamp (Keeler Ophthalmic Instruments, UK). A Frequency Doubling Technology (FDT) perimeter (Zeiss Humphrey Systems & Welch Allyn, USA) was used to assess visual fields. Two 45-degree, colour fundus photographs were taken, centred on the optic disc and the macula, respectively, using a Digital Retinography System (DRS) non-mydriatic fundus camera (CenterVue SpA, Italy). Tropicamide (0.5%) was used to induce mydriasis when image quality was reduced due to small pupil size, and photographs were taken again. The DRS camera was also used to take anterior segment photographs for those with vision loss in one or both eyes. Intraocular pressure was measured using a tonometer (iCare, Finland).

### Identification of eye disease

The presence or absence of the major blinding eye diseases, cataract, AMD, DR and glaucoma were determined in participants with either unilateral or bilateral vision loss, regardless of whether any of these conditions were determined to be the primary cause of vision loss. Blinded retinal graders, an optometrist and ophthalmologists graded all retinal images using OpenClinica software (OpenClinica LLC and collaborators, Waltham, MA, USA) and eye diseases including AMD, DR and glaucoma were graded according to protocols that have been described in detail elsewhere^22–24^. Anterior segment photographs and fundus photographs were assessed to identify participants with cataracts. In cases where photographs were unavailable, cataracts were identified by using a hand-held slit-lamp.

### Statistical analysis

Descriptive statistics including age, number of years of education (continuous variables), gender, language, country of birth, distribution in each remoteness stratum, and time since last eye examination (categorical variables) were calculated for Indigenous and non-Indigenous participants separately. Analyses were performed separately for: (1) those with unilateral vision loss; (2) those with bilateral vision loss and; (3) all those with unilateral and bilateral vision loss combined (the latter group hereafter referred to as *vision loss*). Vision loss was defined as presenting visual acuity worse than 6/12. Due to the stratification of our sample by Indigenous status, reliability and validity analyses were conducted separately for Indigenous and non-Indigenous participants. For each of the major diseases (cataract, DR, AMD, and glaucoma), the numbers of participants with vision loss who (1) correctly reported not having the condition (true negative); (2) incorrectly reported not having the condition (false negative); (3) correctly reported having the condition (true positive); and (4) incorrectly reported having the condition (false positive) were calculated. Reliability of self-report was calculated by deriving Cohen’s Kappa coefficients (κ) and confidence intervals. Validity of self-report was quantified by determining sensitivity, specificity, positive predictive values (PPV), and negative predictive values (NPV) for each eye disease. Reliability and validity were ascertained for participants with unilateral vision loss and bilateral vision loss both separately and in combination.

Univariate and multivariable logistic regression analysis was conducted to identify risk factors associated with unawareness of disease by comparing false negative cases with true positive controls. Logistic regression was conducted for AMD, DR and cataract separately. Regression analysis could not be conducted for glaucoma due to insufficient sample sizes. Risk factors investigated in logistic regression included: age, gender, years of education, English spoken at home, Indigenous status, ethnicity of non-Indigenous participants (Oceanian, European, or Other), geographic remoteness, time since last eye examination (categories were ≤1 year, >1–2 years, >2–5 years, >5 years or never). Risk was expressed as odds ratio (OR) and 95% confidence intervals (CI). Analysis was performed by using Stata, version 14.2 (Stata Corp, College Station, TX).

## Results

### Demographic characteristics

In total, 1738 Indigenous Australians and 3098 non-Indigenous Australians were recruited and examined between the 11^th^ of March 2015 and the 18th of April 2016 from all levels of geographic remoteness in Australia. Of these, 250 (14.4%) Indigenous Australians and 501 (16.2%) non-Indigenous Australians had unilateral vision loss, and a further 188 (10.8%) and 208 (6.7%) had bilateral vision loss, respectively (presenting visual acuity worse than 6/12). The sample of Indigenous Australians with vision loss had a mean [SD] age of 59.6 [10.5] years and 42% were male (Table 1). The mean [SD] age of non-Indigenous Australians with vision loss was 70.5 [10.1] years and 51.9% were male. More than half of participants in both groups reported having undergone an eye examination within the past year. Due to a combination of poor image quality and missing data, the presence or absence of disease could not be determined for cataract in 19% of participants, AMD in 11% of participants and DR in 11% of participants.

**Table 1:** Demographic characteristics of Indigenous and non-Indigenous Australians with unilateral or bilateral vision loss in the National Eye Health Survey.

	Indigenous (n = 438)	Non-Indigenous (n = 709)
Mean years of age (SD)^†^	59.6 (10.5)	70.5 (10.1)
Mean years of education (SD)	10.1 (3.3)	11.8 (3.8)
	*n* (%)	*n* (%)
Gender (male)	184 (42.0)	368 (51.9)
English at home	413 (94.3)	659 (93.0)
*Ethnicity*		
Oceanian	438 (100.0)	476 (67.1)
European	0	179 (22.3)
Others	0	54 (7.6)
*Remoteness*		
Major City	163 (37.2)	290 (40.9)
Inner Regional	70 (16.0)	125 (17.6)
Outer Regional	131 (29.9)	150 (21.2)
Remote	39 (8.9)	94 (13.3)
Very Remote	35 (7.9)	50 (7.0)
*Years since last eye exam*		
≤1 year	228 (52.4)	412 (58.1)
1 <–≤ 2 years	72 (16.6)	143 (20.2)
2 <–≤ 5 years	64 (14.7)	81 (11.4)
>5 years or never	71 (16.3)	73 (10.3)

^†^Note the lower age limit for inclusion of Indigenous Australians than non-Indigenous Australians.

### Reliability and validity of self-report of eye disease in participants with vision loss

#### Kappa coefficients

With the exception of κ = 0.75 (moderate reliability) for glaucoma in Indigenous participants with unilateral vision loss, the reliability of self-report for all four diseases in both Indigenous and non-Indigenous Australians, with unilateral and bilateral vision loss, was poor (κ = −0.09 to 0.35 for cataract; 0.04 to 0.31 for AMD; 0.11 to 0.39 for DR; 0.20 to 0.35 for glaucoma) (Table 2).

**Table 2:** The reliability of self-report of four leading causes of vision loss by Indigenous and non-Indigenous Australians with vision loss

		True positive^†^ *n*	False negative *n*	False positive *n*	True negative *n*	Kappa coefficient (95% CI)	Sensitivity (%) (95% CI)	Specificity (%) (95% CI)	PPV (%) (95% CI)	**NPV (%) (95% CI)**
**Participants with bilateral vision loss (worse than 6/12) (n = 396)**										
Indigenous	Cataract	29	60	38	61	−0.06 (−0.20, 0.08)	32.6 (23.0, 43.3)	61.6 (51.3, 71.2)	43.3 (31.2, 56.0)	50.4 (41.2, 59.6)
	AMD	2	35	3	115	0.04 (−0.07, 0.15)	5.4 (0.7, 18.2)	97.5 (92.7, 99.5)	40.0 (5.3, 85.3)	76.7 (69.1, 83.2)
	Diabetic retinopathy	19	33	3	101	0.39 (0.25, 0.54)	36.5 (23.6, 51.0)	97.1 (91.8, 99.4)	86.4 (65.1, 97.1)	75.4 (67.2, 82.4)
	Glaucoma	3	12	6	167	0.20 (−0.03, 0.44)	20.0 (4.33, 48.1)	96.5 (92.6, 98.7)	33.3 (7.5, 70.1)	93.3 (88.6, 96.5)
Non-Indigenous	Cataract	38	51	62	57	−0.09 (−0.23, −0.04)	42.7 (32.3, 53.6)	47.9 (38.7, 57.2)	38.0 (28.5, 48.3)	52.8 (42.9, 62.5)
	AMD	22	45	6	103	0.31 (0.18, 0.44)	32.8 (21.8, 45.4)	94.5 (88.4, 98)	78.6 (59.0, 91.7)	69.6 (61.5, 76.9)
	Diabetic retinopathy	3	29	3	135	0.11 (−0.04, 0.25)	9.4 (2.0, 25.0)	97.8 (93.8, 99.5)	50.0 (11.8, 88.2)	82.3 (75.6, 87.8)
	Glaucoma	5	7	11	185	0.31 (0.08, 0.55)	41.7 (15.2, 72.3)	94.4 (90.2, 97.2)	31.3 (11.0, 58.7)	96.4 (92.6, 98.5)
**Participants with unilateral vision loss (worse than 6/12) (n = 751)**										
Indigenous	Cataract	39	75	9	67	0.19 (0.09, 0.30)	34.2 (25.6, 43.7)	88.2 (78.7, 94.4)	81.3 (67.4, 91.1)	47.2 (38.8, 55.7)
	AMD	5	50	7	166	0.07 (−0.04, 0.18)	9.1 (3.0, 20.0)	96.0 (91.8, 98.4)	41.7 (15.2, 72.3)	76.9 (70.6, 82.3)
	Diabetic retinopathy	18	45	3	166	0.34 (0.21, 0.47)	28.6 (17.9, 41.3)	98.2 (94.9, 99.6)	85.7 (63.7, 97.0)	78.7 (72.5, 84.0)
	Glaucoma	3	0	2	245	0.75 (0.40, 100)	100 (29.2, 100.0)	99.2 (97.1, 99.9)	60.0 (14.7, 94.7)	100 (98.5, 100.0)
Non-Indigenous	Cataract	114	142	13	76	0.20 (0.13, 0.28)	44.5 (38.3, 50.8)	85.4 (76.3, 92.0)	89.8 (83.1, 94.4)	34.9 (28.6, 41.6)
	AMD	29	125	10	296	0.19 (0.11, 0.27)	18.8 (13.0, 25.9)	96.7 (94.1, 98.4)	74.4 (57.9, 87.0)	70.3 (65.7, 74.6)
	Diabetic retinopathy	6	57	1	394	0.15 (0.04, 0.26)	9.5 (3.6, 19.6)	99.7 (98.6, 100)	85.7 (42.1, 99.6)	87.4 (83.9, 90.3)
	Glaucoma	7	4	32	458	0.25 (0.09, 0.42)	63.6 (30.8, 89.1)	93.5 (90.9, 95.5)	17.9 (7.5, 33.5)	99.1 (97.8, 99.8)
**Participants with either bilateral or unilateral vision loss (worse than 6/12) (n = 1147)**										
Indigenous	Cataract	68	135	47	128	0.06 (−0.03, 0.15)	33.5 (27.0, 40.4)	73.1 (65.9, 79.6)	59.1 (49.6, 68.2)	48.7 (42.5, 54.9)
	AMD	7	85	10	281	0.06 (−0.02, 0.14)	7.6 (3.1, 15.1)	96.6 (93.8, 98.3)	41.2 (18.4, 67.1)	76.8 (72.1, 67.1)
	Diabetic retinopathy	37	78	6	267	0.37 (0.27, 0.46)	32.2 (23.8, 41.5)	97.8 (95.3, 99.2)	86.0 (72.1, 94.7)	77.4 (72.6, 81.7)
	Glaucoma	6	12	8	412	0.35 (0.13, 0.57)	33.3 (13.3, 59.0)	98.1 (96.3, 99.2)	42.9 (17.7, 71.1)	97.2 (95.1, 98.5)
Non-Indigenous	Cataract	152	193	75	133	0.07 (−0.003, 0.15)	44.1 (38.7, 49.5)	63.9 (57.0, 70.5)	67.0 (60.4, 73.0)	40.8 (35.4, 46.3)
	AMD	51	170	16	399	0.23 (0.16, 0.30)	23.1 (17.7, 29.2)	96.1 (93.8, 97.8)	76.1 (64.1, 85.7)	70.1 (66.2, 73.9)
	Diabetic retinopathy	9	86	4	529	0.14 (0.05, 0.22)	9.5 (4.4, 17.2)	99.2 (98.1, 99.8)	69.2 (38.6, 90.9)	86.0 (83.0, 88.7)
	Glaucoma	12	11	43	643	0.28 (0.14, 0.41)	52.2 (30.6, 73.2)	93.7 (91.6, 95.4)	21.8 (11.8, 35.0)	98.3 (97.0, 99.2)

^†^True positive: self-report = yes, examination = yes; False negative: self-report = no, examination = yes; False positive: self-report = yes, examination = no; True negative: self-report = no, examination = no 95% CI = 95% Confidence Interval. PPV = Positive Predictive Value. NPV = Negative predictive value. AMD = Age-related macular degeneration.

The κ coefficients for cataract were similar for both Indigenous and non-Indigenous Australians (bilateral vision loss = −0.06 vs −0.09 and unilateral vision loss = 0.19 vs 0.20), however, κ for AMD was lower for Indigenous Australians (bilateral vision loss = 0.04 vs 0.31 and unilateral vision loss = 0.07 vs 0.19) while DR was higher for Indigenous Australians (bilateral vision loss = 0.39 vs 0.11 and unilateral vision loss = 0.34 vs 0.15). κ for glaucoma was higher for non-Indigenous Australians with bilateral vision loss (0.31 vs 0.20), but lower for non-Indigenous Australians with unilateral vision loss (0.75 vs 0.25).

#### Sensitivities and specificities

Sensitivities were almost uniformly low for all eye diseases, with the exception of three Indigenous participants with unilateral vision loss and glaucoma (100% sensitivity) (Table 2). Sensitivities for glaucoma (Indigenous Australians = 33% and non-Indigenous Australians = 52%) and cataract (Indigenous Australians = 33% and non-Indigenous Australians = 45%) were comparably poor. The lowest sensitivities were found for Indigenous Australians with AMD and non-Indigenous Australians with DR. Only 5.4% of bilaterally-impaired and 9.1% of unilaterally-impaired Indigenous Australians with AMD reported that they had the disease. Only 9.4% and 9.5% of non-Indigenous Australians with DR and bilateral or unilateral vision loss, respectively, were aware that they had the disease. Between 93% and 99.7% of participants who were clinically identified as not having AMD, DR and glaucoma correctly self-reported that they did not have each disease, suggesting high specificity for all three diseases. Conversely, specificity for cataract was variable, being low in those with bilateral vision loss (62% for Indigenous and 48% for non-Indigenous Australians), and moderately high in those with unilateral vision loss (88% and 85%, respectively).

#### Positive Predictive Values and Negative Predictive Values

PPVs for glaucoma were consistently low, with fewer than half (43%) of Indigenous and only one fifth (22%) of non-Indigenous Australians who reported having the disease being diagnosed with glaucoma in this study (Table 2). While PPVs were below 50% for cataract in those with bilateral vision loss, they were moderately high for both Indigenous and non-Indigenous participants with unilateral vision loss (PPV = 81% and 90%). Most Indigenous Australians who self-reported DR were confirmed to have the condition (PPV = 86%), which was considerably higher than 69% for non-Indigenous Australians. Approximately 40% of Indigenous Australians and 76% of non-Indigenous Australians who self-reported AMD were identified as having the condition. Between 93% and 100% of participants who self-reported that they did not have glaucoma were determined to not have the disease, while NPVs for both AMD and DR were lower, with 70–87% accurately reporting that they did not have each disease. NPVs were lowest for participants reporting that they did not have cataracts, with between only one third and one half of those reporting no history of cataracts being cataract-free.

#### Risk factors for unawareness of eye disease

Multivariable logistic regression revealed that non-Indigenous Australians were less likely to be aware that they had DR than their Indigenous counterparts (OR 0.25) (Table 3). Conversely, Indigenous Australians were at greater risk than non-Indigenous Australians of being unaware that they had cataracts (OR 1.56) and AMD (OR 3.64) in univariate analysis, but this association was not significant in multivariable analysis. Older age was associated with unawareness of AMD (OR 0.89/year) and cataract (0.95/year), but not of DR. Participants with cataracts were at significantly greater risk of being unaware of their cataracts if their last eye examination occurred more than one year earlier (p < 0.001 for all time categories measured). While time since last eye examination was not a statistically significant independent risk factor for unawareness of AMD (p = 0.061 for 1–2 years) and DR (p = 0.067 for more than 5 years) in multivariable analysis, univariate analysis suggested a significantly greater risk of disease unawareness with longer times since the last examination (ORs 3.10 for 2–5 years and 3.46 for 1–2 years compared to less than 1 year for AMD and 6.44 for 1–2 years and 11.11 for 5 or more years compared to less than 1 year for DR). For all three diseases, participants who had visited an ophthalmologist for their last eye examination were significantly more likely to be aware of their eye diseases than those who had visited an optometrist (p = 0.002 to p < 0.001).

**Table 3:** Independent risk factors associated with unawareness of eye disease by cause.

Factor	AMD (n = 313)		Diabetic retinopathy (n = 210)		Cataract (n = 548)	
	OR (95% CI)	p	OR (95% CI)	p	OR (95% CI)	p
Indigenous status			0.25 (0.10, 0.65)	0.004		
Age (year)	0.89 (0.84, 0.93)	<0.001			0.95 (0.93, 0.97)	<0.001
***Time since last eye test***						
≤1 year					1	
1 <–≤ 2 years					2.62 (1.55, 4.42)	<0.001
2 <–≤ 5 years					4.33 (2.27, 8.23)	<0.001
>5 years or never					28.96 (6.09, 137)	<0.001
***Service provider last used***						
Optometrist	1		1		1	
Ophthalmologist	0.32 (0.16, 0.64)	0.001	0.19 (0.08, 0.44)	<0.001	0.47 (0.30, 0.75)	0.002

OR = Odds ratio. AMD = Age-related macular degeneration Only risk factors that were shown to have independent statistically significant associations (p < 0.05) in multivariable logistic regression analysis are shown. Other factors tested included; gender, years of education, English spoken at home, ethnicity of non-Indigenous Australians, and geographic remoteness.

## Discussion

This paper examined the reliability and validity of self-report of eye disease in participants with vision loss in Australia’s first National Eye Health Survey. To our knowledge, this is the first Australian study to conduct a thorough analysis on agreement between self-report and clinical diagnosis of the four major eye diseases, AMD, DR, cataract and glaucoma. Reliability of self-report was mostly poor for all eye diseases, in both Indigenous and non-Indigenous Australians, with both unilateral and bilateral vision loss. While measures of validity were variable, with mostly high disease specificities and moderate to high predictive values in some instances, the validity of self-report was generally shown to be poor. These results illustrate that self-report of major eye diseases, even in those with vision loss, is an unreliable indicator of the presence or absence of eye disease in the Australian population. The results of surveys such as the NHS and NSDAC that rely on self-report to determine the prevalence of eye disease should therefore be interpreted with great caution.

Sensitivity of self-report by both Indigenous and non-Indigenous Australians was consistently low for all diseases, with the exception of Indigenous Australians with unilateral vision loss and glaucoma. It should be noted that the number of participants identified with glaucoma was significantly lower than for other diseases, and this small sample size necessitates cautious inference about the wider population. Nonetheless, as few as 8% of Indigenous Australians with AMD, 10% of non-Indigenous Australians with DR, and approximately one third of all participants with cataract were aware of their eye condition, illustrating that self-report of eye disease may dramatically under-estimate true disease prevalence in the population. While still low, the sensitivity for DR in Indigenous Australians (32%) was 3.5 times higher than for non-Indigenous Australians, and this difference was found to be highly significant in logistic regression (p = 0.004). With a PPV of 86%, Indigenous Australians who reported that they had DR were more likely to be correct than for all other eye diseases measured. Together, these results signify that increases in uptake of Indigenous Australians to DR screening services, as well as improvements in education and coordinated outreach initiatives to Indigenous communities may be modestly improving disease awareness in the Indigenous population^25, 26^. Interestingly, higher sensitivities and PPVs for AMD in non-Indigenous Australians than their Indigenous counterparts may reflect better knowledge and literacy of the condition in this group, possibly reflecting the higher prevalence of AMD in the non-Indigenous population^27^. As we did not have access to participants’ medical records, it was not possible to ascertain the proportion of disease unawareness that was due to participants having never been diagnosed versus the proportion who had been diagnosed but failed to recall their eye diseases.

Specificity of self-report was consistently high (above 90%) for AMD, DR and glaucoma, suggesting that participants without each disease were generally able to report that they were disease-free. While high specificities are generally favourable, in this instance when considered in conjunction with very low sensitivities and low to moderate NPVs, these high specificities may result from a cognitive bias where the overwhelming proportion of individuals provide a ‘no’ self-report due to potential uncertainty, regardless of their true disease status^28^. Conversely, self-report for cataract was characterised by higher sensitivities and lower specificities than the other diseases. This may reflect a propensity for older Australians with vision loss to more readily attribute their vision loss to cataract over other diseases, perhaps owing to cataract being a better-known and more common disease^10^.

As literature on the validity of self-report of eye disease is scarce, and existing studies employed different statistical measures or investigated population sub-groups that differed significantly from the present study^7, 8, 11, 16^, comparison between studies is difficult. The National Indigenous Eye Health Survey (NIEHS) reported PPVs for self-report of eye diseases in Indigenous Australians, ranging from 8.7% for AMD to 48% for cataract^16^, all of which were substantially lower than those in the present study, suggesting that improvements in eye health education in Indigenous communities in recent years may have slightly improved the ability of Indigenous Australians to correctly identify their eye diseases^29^. Overall sensitivities for self-report of eye disease in the NEHS and the BDES were comparably low, with the BDES reporting sensitivities of 38% and 18% for cataract and AMD^8^, and the NEHS reporting sensitivities of 34% to 44% for cataract and 7.6% to 23% for AMD. Even though the BDES findings relate to all members of the study, the current study focused only on those with vision loss, and yet overall agreement as ascertained by κ was substantially lower in the NEHS (0.06 to 0.37) than in the BDES (0.50 to 0.78). The low sensitivity and high specificity of self-report of glaucoma in our study are similar to those of the Melbourne VIP, which was conducted more than twenty years ago^18^. However, as our sample included only those with vision loss, validity of self-report for glaucoma is likely to be even poorer in the general population, and therefore lower than that identified in the Melbourne VIP. The poor reliability of self-report in this Australian sample points either to poor eye health literacy or low rates of disease detection and necessitates an intensive public health campaign to increase awareness of eye diseases.

This paper identified risk factors associated with false negative self-reports of eye disease. Those examined more than five years ago were 29 times more likely to be unaware of their cataracts, strongly emphasising the importance of regular eye examinations. Considering that cataract is the second leading cause of reversible vision loss in Australia^27, 30^, and that approximately two-thirds of participants were unaware of their cataracts, these individuals represent a substantial proportion of Australians with reversible vision loss. Improving self-awareness of disease status in these individuals through education and increased frequency of eye examinations may increase cataract extraction rates and reduce the prevalence of vision loss in Australia. Participants with DR, AMD and cataract who had visited an ophthalmologist were more likely to be aware of their diseases than those who had seen an optometrist, perhaps owing to the fact that those attending ophthalmology clinics may be more likely to have more advanced and symptomatic disease. Lack of disease awareness in those with early stage disease may be mitigated by increasing the frequency of eye examinations and improving referral pathways to ophthalmology services, consequently improving the timely treatment of disease^31^.

It is important to note that most of the risk factors measured, including gender, level of education, spoken language, ethnicity, and geographic remoteness, were not significantly associated with a greater risk of inaccurate self-report. Considered in conjunction with the almost uniformly poor reliability of self-report shown in other studies^7, 8, 16, 18^, the inaccuracy across most sociodemographic variables in the NEHS, reaffirms that self-report is a universally unreliable measure of the prevalence of major eye diseases. However, the reliability of the risk factor analysis conducted in this survey is somewhat limited and must be considered with caution. Sociodemographic and eye healthcare utilisation data were obtained using self-report. As we have illustrated, the reliability of self-report of eye disease is unreliable, and we therefore cannot rule out inherent inaccuracies in other data collected using self-report. Another limitation of this study was that disease status could not be definitively determined for some participants (19% for cataract, 11% for AMD and 11% for DR). These individuals could not be included in the agreement analysis, and there is therefore a small but unquantifiable risk of over- or under-estimation of agreement between self-report and diagnosed eye disease in this study.

In conclusion, the generally poor reliability of self-report of eye diseases has significant implications for eye healthcare policy and disease surveillance based on this method of data collection. Our findings have demonstrated that, even in a population with vision loss, self-report of eye disease is highly inaccurate, and that surveys relying on self-report are unlikely to generate accurate or representative information on the prevalence of eye disease in the population. Population surveys aiming to collect accurate data on the prevalence of vision-threatening eye disease must include a standardised clinical eye examination protocol. Furthermore, individuals who are unaware of their eye diseases are at particularly high risk of vision loss^9^. Improving eye disease literacy and the frequency of eye examinations in older Australians may improve disease awareness and treatment, thereby reducing Australia’s burden of vision impairment and blindness.

### Data availability statement

The Royal Victorian Eye and Ear Hospital Human Research Ethics Committee and the numerous state-level Indigenous Ethics bodies have placed stringent ethical guidelines on the investigators of this study. Due to the risk of identifying participants, particularly in remote Indigenous communities, the authors are unable to make the dataset freely available. Interested researchers may contact the Principal Investigator, Dr.Mohamed Dirani, Head of Evaluative Research and Health Services, Centre for Eye Research Australia at mdirani@unimelb.edu.au to request access to the data.
